# Kidney Paired Donation (KPD) in Brazil: first 3-way case report

**DOI:** 10.1590/2175-8239-JBN-2025-0177en

**Published:** 2026-03-06

**Authors:** Juliana Bastos, Glaucio Silva de Souza, Marcio Luiz de Sousa, Pedro Bastos Guimarães de Almeida, Thais Freesz, David Jose de Barros Machado, Elias David-Neto, Gustavo Fernandes Ferreira

**Affiliations:** 1 Santa Casa de Misericórdia de Juiz de Fora, Unidade de Transplante, Juiz de Fora, MG, Brazil.; 2 Universidade de São Paulo, Faculdade de Medicina, Hospital das Clínicas, Unidade de Transplante Renal, São Paulo, SP, Brazil.

**Keywords:** Kidney Paired Donation, Living Kidney Donor Transplantation, Chronic Kidney Disease, Transplantation Logistics in Brazil, Immunologic Incompatibility

## Abstract

Kidney Paired Donation (KPD) is a transformative strategy in living kidney donor transplantation (LDKT), particularly for overcoming immunological barriers that preclude direct donation. In 2021, KPD accounted for one-fifth of adult LDKT and for half of LDKT for sensitized recipients in the United States. In Brazil, with a high prevalence of chronic kidney disease (CKD) and over 30,000 patients on transplant waiting lists, the demand for compatible donors far exceeds supply. This article presents a case report of KPD in the Brazilian context, illustrating its feasibility and highlighting challenges and considerations for broader implementation. The case demonstrates KPD’s potential to increase transplant rates, improve outcomes, and reduce dialysis costs. Nevertheless, structural, ethical, and regulatory challenges remain. This report emphasizes the implications of expanding KPD as a sustainable, life-saving strategy in Brazil.

## Introduction

In the United States, both the Kidney Allocation System (KAS) and Kidney Paired Donation (KPD) programs have contributed to increasing transplantation rates among highly sensitized recipients, with KPD offering an important pathway to living-donor kidney transplantation (LDKT) in this group^1,2^. In 2021, one-fifth of adult LDKT and half of LDKT for sensitized recipients were facilitated by KPD^1^. The incidence of chronic kidney disease (CKD) continues to rise globally, affecting an estimated 10% of the population and resulting in 1.2 million deaths annually^3,4^. The 2022 Dialysis Census in Brazil reported 153,831 patients undergoing dialysis, of which the public health system financed 80.2%. The annual mortality rate among these patients ranged from 17–20% in the past five years^5^.

Kidney transplantation is the optimal treatment for end-stage CKD, offering superior quality of life and survival rates compared with dialysis. Despite these advantages, only 6,047 kidney transplants were performed in Brazil in 2023, with only 16.5% from living donors, leaving over 32,000 patients waiting for transplants^6^. This number represents less than half of the estimated demand, highlighting Brazil’s limited access to kidney transplantation. LDKT provides better patient and graft survival outcomes than deceased-donor transplants, significantly improving patient longevity and quality of life^7,8^. Furthermore, it has been well established that LDKT is cost-effective, offering substantial savings over dialysis^9,10^. Within this context, KPD represents a complementary strategy to expand access to LDKT and improve opportunities for compatible matches, particularly among immunologically incompatible pairs, as part of broader efforts to reduce the kidney transplant waitlist in Brazil.

Paradoxically, Brazil’s rate of living donor transplants has been declining, exacerbated by the COVID-19 pandemic^6^. While individuals up to the fourth degree of kinship or spouses are eligible to be living donors, those with more distant relationships or unrelated donors require judicial approval^11^. However, these restrictions do not affect transplant success, as studies have shown similar outcomes between related and unrelated pairs^9,12^. A significant barrier to LDKT is immunologic incompatibility, such as ABO incompatibility (ABOi) and positive crossmatch (PC+), disqualifying approximately 30–50% of potential donors^7,13^.

KPD has been conceptualized to overcome these barriers. First proposed by Rapaport in 1986, KPD enables incompatible donor-recipient pairs to exchange donors, thus allowing transplants that would otherwise be impossible^14^. This approach has since expanded worldwide, with programs established in Europe, the United States, Canada, Australia, and other regions^15,16,17^. Only a few countries in Latin America, including Brazil, have reported isolated KPD cases, and Mexico appears to be the only country with an active program^18,19,20^. By allowing longer chains and complex exchanges, KPD can significantly increase transplant rates, benefiting sensitized patients who would not otherwise find compatible donors.

Due to the characteristics of recipients enrolled in KPD programs, it is not uncommon for exchanges involving only two pairs to be insufficient to facilitate transplantation. Longer chains are often necessary to accommodate highly sensitized patients. In 2006, it was reported that a KPD program increased its transplantation capacity from 17.7% to 24.4% by allowing exchanges involving three pairs rather than limiting them to only two pairs^21^. In 2010, it was demonstrated that a program permitting exchanges with N pairs could increase its transplantation capacity by 18%, while raising the average panel reactive antibody (PRA) score of transplanted recipients by 9%, compared to a program restricting two-pair exchanges^22^. This innovative solution holds great promise for Brazil’s transplant landscape^23^; however, establishing a national KPD program would require significant logistical and regulatory efforts.

Brazil has seen isolated cases of KPD but lacks a structured national program. This report describes the first three-pair kidney paired donation performed in Brazil at *Santa Casa de Misericórdia de Juiz de Fora* (SCMJF), representing the initial clinical experience with this modality in the country’s transplant system. This case demonstrates KPD’s potential to increase transplant rates and reduce healthcare costs while highlighting the logistical, regulatory, and ethical challenges that must be addressed for broader implementation. The success of this pioneering three-way exchange underscores the viability of KPD as a sustainable solution to the kidney donor shortage in Brazil, setting the stage for further development of a potential national KPD program.

### Ethics

The research project was initially approved by the Research Ethics Committee of the *Hospital das Clínicas* at the University of São Paulo (HCFMUSP) in 2018 as a single-center study (CAAE: 83469518.4.0000.0068). In 2022, an amendment was approved by the same ethics committee, expanding it to a multicenter study (CAAE: 83469518.4.0000.0068), and by the Ethics Committee of *Santa Casa de Misericórdia de Juiz de Fora* (SCMJF) (CAAE: 83469518.4.2001.5139). This case report was also approved by the Research Ethics Committee of SCMJF, in accordance with national and international ethical guidelines for research involving human subjects (CAAE: 93197825.0.0000.5139).

Since September 2022, through a non-profit partnership with the Alliance for Paired Kidney Donation, the KidneyMatchGrid tool has been used to include recipient-donor pairs and conduct match runs (in which the tool searches for possible matches among registered pairs). In December 2023, the possibility of an exchange involving three pairs from the same institution (SCMJF) was identified.

The project complied with all Brazilian legal requirements, following a sequential approval process that included Ethics Committee authorization, judicial approval, and subsequent authorization from both the State and National Transplant Systems. The judicial process was conducted by a lawyer affiliated with and funded by the project, who managed all legal procedures in a way that prevented any contact between the participating pairs.

### Original Pairs (Table 1)

**Table 1:** Demographic Data

Donors			
	D1	D2	D3
Sex	Female	Male	Female
Ethnicity	White	White	Mixed-race
Age	48	47	57
BMI	28.5	27.9	25.0
Blood Type	A	A	O
eGFR	92	141	119
Relation	Daughter	Husband	Sister
Recipients			
	R1	R2	R3
Sex	Male	Female	Female
Ethnicity	Mixed-race	Mixed-race	Mixed-race
Age	69	44	53
BMI	18.4	21.9	17.2
Blood Type	O	A	A
cPRA	00%	69.5%	93.8%
First Tx	Yes	Yes	No
Kidney disease	Hypertension	Unknown	Unknown
Dialysis vintage (m)	10	18	12

Recipient 1 (R1), male, 69 years old, on hemodialysis (HD) since January 2023 due to hypertensive nephropathy, blood type O. He was listed for deceased donor kidney transplant since October 2023, with a PRA of 0% for both class I and II. His daughter, donor 1 (D1), 48 years old, blood type A, was a candidate for donation but was incompatible with ABOi. The R1–D1 pair was enrolled in the KPD program in November 2023.

Recipient 2 (R2), female, 44 years old, has been on HD since August 2022 because of CKD of indeterminate etiology, blood type A. She was listed for deceased donor transplant since January 2023, with a calculated PRA (cPRA) of 69.5%. Her husband, donor 2 (D2), 47 years old, blood type A, was a candidate for donation but was contraindicated due to PC+. The R2–D2 pair was enrolled in the KPD program in January 2023.

Recipient 3 (R3), female, 53 years old, has been on HD since August 2022 due to CKD of indeterminate etiology, blood type A. She had undergone LDKT between 2004 and 2022. She was listed for transplantation in June 2023, with a cPRA of 93.7%. Her sister, donor 3 (D3), 57 years old, blood type O, was contraindicated due to PC+. The R3–D3 pair was enrolled in the KPD program in September 2023 (Table 1).

### Matching

**Figure 1:**
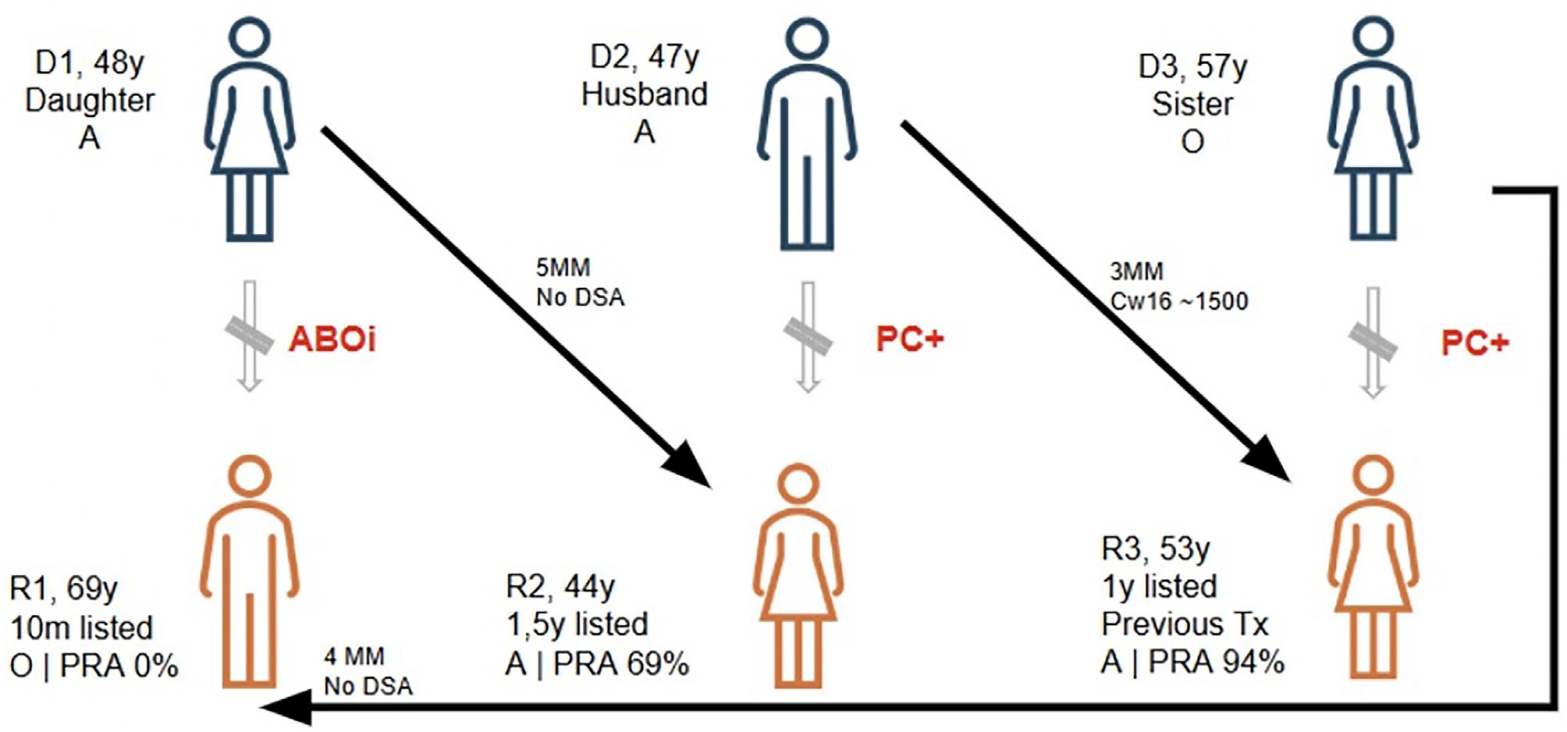
Three-way paired kidney transplantation.

The KidneyMatchGrid tool identified compatibility between D1 and R2, D2 and R3, and D3 and R1 (Figure 1). The degree of HLA mismatching among the paired exchanges ranged from three to five *loci* (mean of four), representing acceptable compatibility for proceeding with transplantation. The compatibility was further confirmed by a negative flow cytometry crossmatch. Despite the sensitized profiles of the involved recipients, only R3 presented a donor-specific antibody (DSA) identified as Cw16 with an MFI of 1584 in the current serum and DQ8 with an MFI of 1628 in the historical serum. Additionally, none of the pairs identified had donor–recipient infection-risk mismatches.

As described above, the project was approved by the ethics committees of both hospitals. When a potential match was identified, authorization was requested first from the hospital’s Ethics Committee, followed by judicial approval, which is required for non-related donors under Brazilian law, and then from the state and national transplant authorities. This bureaucratic process is time-consuming, highlighting one of the main challenges for the practical implementation of KPD programs. Legal changes—such as the bill currently under consideration in the Brazilian Chamber of Deputies^24^—appear to be essential to enable the establishment of an effective national KPD program.

### Surgery

All six surgeries were performed on August 10, 2024, at SCMJF. Six operating rooms were used simultaneously, with donor surgeries starting first, immediately followed by the corresponding recipient procedures. Each team included four surgeons, anesthesiologists, and surgical residents. Such simultaneous operations require substantial personnel, coordination, and hospital infrastructure. Discussion of desynchronized scheduling for larger chains is beyond the scope of this study.

Anonymity was maintained before and during all stages of the paired donation process. Pre-transplant evaluations and laboratory tests were scheduled separately to prevent contact between participants. On the day of surgery, donors and recipients were admitted to different hospital floors and transferred to the operating room separately. Participants were informed that any post-transplant meeting would occur only with mutual consent, as specified in the informed consent form. With authorization from all parties, introductions took place after the procedures.

The procedures followed standard institutional practices and were completed without complications. Recipients received induction therapy with thymoglobulin at 2.25 mg/kg, except for the recipient with DSA, who received 6 mg/kg. Maintenance immunosuppression consisted of tacrolimus, sirolimus, and prednisone for all patients.

**Figure 2:**
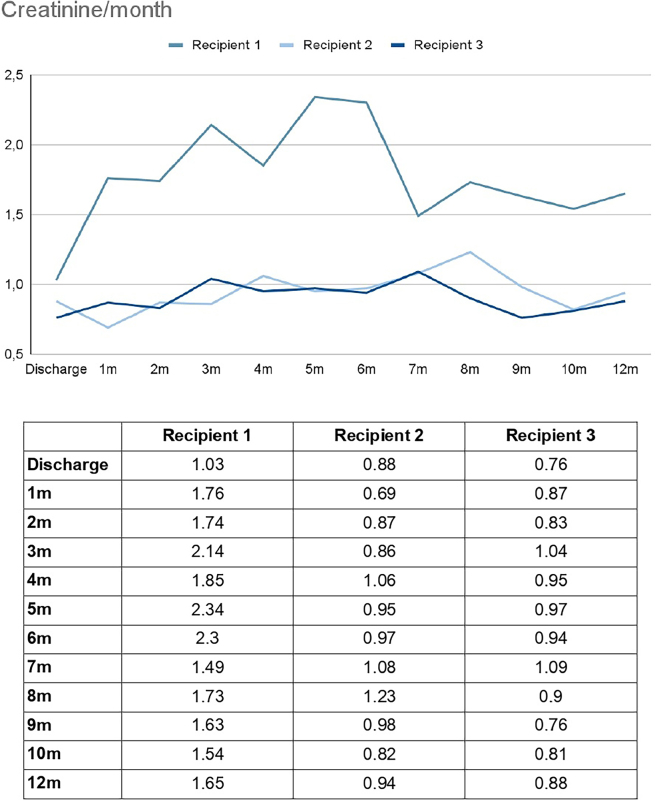
Recipients creatinine (mg/dL).

According to institutional criteria, donor eligibility required a creatinine clearance above 80 mL/min, normal urinalysis, and absence of comorbidities or anatomical contraindications. All donors were discharged on postoperative day (POD) 2, and all recipients on POD 4. Every recipient demonstrated immediate graft function, with serum creatinine levels ranging from 0.8 to 1.0 mg/dL at discharge. All recipients demonstrated excellent graft function at one year, as illustrated in Figure 2. It is important to note that R1 experienced a transient decline in renal function due to a prostatic condition, unrelated to the donor, and likely achieved good outcomes after resolution because of the high quality of the living-donor kidney. He also developed post-transplant diabetes mellitus. Aside from this, there were no other hospitalizations or major complications among the recipients. None of the patients showed suspicion of rejection or required a biopsy for any reason. Additionally, R3 had DSA monitored, which remained at MFI < 1000 throughout follow-up.

Importantly, all donors also showed exceptional postoperative recovery, with no complications during the first year after donation. All maintained satisfactory renal function, with estimated glomerular filtration rate (eGFR) values ranging from 58 to 83 mL/min.

## Discussion

The first experience with KPD in Brazil occurred in March 2020 at HCFMUSP, involving an exchange between two ABOi pairs, which was successfully performed^19^. In 2022, we published an article discussing the potential benefits of KPD and proposed that the topic be brought forward for broader discussion within society^25^. The editorial supported the benefits of KPD, backed by the existing literature, emphasizing that little to no social or political consequences have been observed from KPD programs worldwide. Given that kidney transplantation is a superior therapy for treating CKD, paired donation should be considered for all patients with potential but incompatible donors^26^.

At the American Transplant Congress in 2024, we presented a survey of 89 living donation candidates at the SCMJF, showing that 70% were unaware of KPD, yet 80% expressed willingness to participate in the program^27^. The cost of dialysis in Brazil is extremely high^28^, with kidney transplantation being the most cost-effective treatment, in addition to its other known benefits. LDKT provides better survival rates than deceased donor kidney transplants (DDKT), making LDKT more economically advantageous^16,29^. A national study published in 2016 estimated that DDKT generates per-patient savings of R$37,000 to R$74,000 compared to HD and PD, respectively, whereas LDKT results in even more significant savings of R$46,000 and R$82,000^30^.

For the successful nationwide implementation of a KPD program, Brazil—similar to other developing countries—must address several structural and regulatory challenges^31^. These include the development of robust logistical infrastructure, the simplification of complex bureaucratic pathways, and the allocation of sustainable financial resources across a country of continental dimensions. The multilayered approval process required for each KPD case remains particularly time-consuming and represents one of the main operational barriers to scaling this initiative. Therefore, legal and regulatory updates—such as the bill currently under discussion in the Brazilian Chamber of Deputies^24^—will likely be essential to enable the establishment of an efficient and permanent national KPD program. Given Brazil’s public and centralized transplant framework, the establishment of a KPD program would only be meaningful if organized as a single national initiative coordinated by the Ministry of Health through the National Transplant System, ensuring equitable access and unified ethical oversight.

For an effective multicenter program, international experience suggests that organ transport is the most efficient approach^32,33^. This allows donors and recipients to remain at their original center under the care of their trusted teams. In Brazil, commercial aviation is routinely used for transporting organs from deceased donors, and we believe that, with proper authorization, this approach would also be optimal for paired-donation programs. However, a detailed discussion of this strategy is beyond the scope of the present study.

Additionally, it is necessary to invest in the technological and human resource development of immunology laboratories. Equally important is the dissemination of accurate information within both the medical community and the general public to dispel misconceptions and prejudices, particularly those related to non-directed and unrelated living kidney donation.

Although only one of the three recipients in this case was classified as highly sensitized, the inclusion of a broader range of donor–recipient pairs aligns with international KPD practices, since maintaining diversity within the pool is crucial to achieving feasible matches and to expanding access for those with significant immunologic challenges. Using a prediction model presented at the World Transplant Congress in 2025^34^, the estimated one-year probability of transplantation for these patients in our institution would have been 40.6% for R1, 35.1% for R2, and only 9.7% for R3. The model predicted an average waiting time of 17 months for R1, 21 months for R2, and no predicted transplantation within 120 months for R3. Through the KPD program, these patients not only achieved the superior outcomes associated with LDKT but also did so in a shorter timeframe. For R3, in particular, participation in the KPD program likely represented her only realistic opportunity for retransplantation.

Currently in Brazil, recipients with a healthy and willing living donor who is immunologically incompatible are left in the same situation as those without a donor, facing limited chances for transplantation. This affects not only recipients who cannot access LDKT and remain on the waitlist, often without real chances of receiving a transplant, but also donors who are unable to fulfill their wish to donate, leaving their loved ones waiting. The KPD optimizes the use of a scarce and valuable resource: healthy and motivated kidney donors. By doing so, KPD has the potential not only to increase the number of transplants and improve graft and recipient survival but also to prevent organ trafficking, an illegal practice opposed by Brazilian society and the transplant community^35,36^.

The main barriers to implementing a national KPD program in Brazil include legal ambiguity, complex logistics, and limited institutional integration across transplant centers. Broader implementation will require legislative reform, national coordination by the Ministry of Health, and sustained investment in training and infrastructure to ensure equity and transparency.

## Data Availability

The datasets supporting the findings of this study are available from the corresponding author, Juliana Bastos, upon reasonable request. The datasets are not publicly available because they contain sensitive clinical data derived from medical records and partial data from an ongoing doctoral research project; however, they may be made available upon justified request to the corresponding author, in accordance with applicable ethical and legal requirements.
