# Supplementary material for: Kidney Paired Donation (KPD) in Brazil: first 3-way case report
Source: J Bras Nefrol. 2026 Mar 6;48(1):e20250177. doi: 10.1590/2175-8239-JBN-2025-0177en (PMC12965706; doi:10.1590/2175-8239-JBN-2025-0177en)
Supplement: Translation

# Translation: Doação Renal Pareada (DRP) no Brasil: relato do primeiro caso envolvendo três duplas

**DOI:** 10.1590/2175-8239-JBN-2025-0177pt

**Authors:** Juliana Bastos, Glaucio Silva de Souza, Marcio Luiz de Sousa, Pedro Bastos Guimarães de Almeida, Thais Freesz, David Jose de Barros Machado, Elias David-Neto, Gustavo Fernandes Ferreira

**Affiliations:** 1 Santa Casa de Misericórdia de Juiz de Fora, Unidade de Transplante, Juiz de Fora, MG, Brasil.; 2 Universidade de São Paulo, Faculdade de Medicina, Hospital das Clínicas, Unidade de Transplante Renal, São Paulo, SP, Brasil.

**Keywords:** Doação Renal Pareada, Transplante Renal com Doador Vivo, Doença Renal Crônica, Logística de Transplantes no Brasil, Incompatibilidade Imunológica

## Abstract

A Doação Renal Pareada (DRP) é uma estratégia transformadora no contexto do Transplante Renal com Doador Vivo (TxRDV), especialmente por superar barreiras imunológicas que impedem a doação direcionada. Em 2021, a DRP correspondeu a um quinto de todos os TxRDV em adultos e à metade dos TxRDV realizados em receptores sensibilizados nos Estados Unidos. No Brasil, com a alta prevalência de Doença Renal Crônica (DRC) e mais de 30 mil pacientes em lista de espera para transplante, a demanda por doadores compatíveis excede amplamente a oferta disponível. Este artigo apresenta um relato de caso de DRP no contexto brasileiro, ilustrando sua viabilidade e destacando os desafios e considerações para sua implementação em maior escala. O caso demonstra o potencial da DRP para aumentar as taxas de transplante, melhorar os desfechos clínicos e reduzir os custos com diálise. Apesar disso, ainda persistem desafios estruturais, éticos e regulatórios. Este relato enfatiza as implicações da expansão da DRP como uma estratégia sustentável, com potencial impacto no acesso ao transplante, na qualidade de vida e na sobrevida dos pacientes no Brasil.

## Introdução

Nos Estados Unidos, tanto o novo sistema de alocação de rins (KAS, do inglês *Kidney Allocation System*) quanto os programas de Doação Renal Pareada (DRP) contribuíram para o aumento das taxas de transplante entre receptores altamente sensibilizados, sendo a DRP uma via essencial para o Transplante Renal com Doador Vivo (TxRDV) nesse grupo^1,2^. Em 2021, um quinto de todos os TxRDV em adultos e metade dos realizados em receptores sensibilizados ocorreram por meio de programas de DRP^1^.

A incidência de Doença Renal Crônica (DRC) continua a aumentar globalmente, afetando cerca de 10% da população e resultando em aproximadamente 1,2 milhão de óbitos anualmente^3,4^. O Censo de Diálise Brasileiro de 2022 relatou 153.831 pacientes em terapia dialítica, dos quais 80,2% são financiados pelo Sistema Único de Saúde (SUS). A mortalidade anual desses pacientes variou entre 17% e 20% nos últimos cinco anos^5^.

O transplante renal é o tratamento padrão-ouro para a DRC terminal, oferecendo melhor qualidade de vida e sobrevida em comparação à diálise. Apesar dessas vantagens, apenas 6.047 transplantes renais foram realizados no Brasil em 2023, sendo 16,5% provenientes de doadores vivos, enquanto mais de 32.000 pacientes permaneciam em lista de espera^6^. Esse número representa menos da metade da demanda estimada, evidenciando o acesso limitado ao transplante renal no país.

O TxRDV proporciona melhores desfechos de sobrevida do paciente e do enxerto quando comparado ao transplante com doador falecido, além de promover maior sobrevida e qualidade de vida^7,8^. Além disso, está bem estabelecido que o TxRDV é custo-efetivo, oferecendo economia substancial em relação à diálise^9,10^. Nesse contexto, a DRP surge como uma estratégia complementar para ampliar o acesso ao TxRDV e aumentar as chances de compatibilidade entre pares incompatíveis, contribuindo, assim, para a redução da lista de espera por transplante renal no Brasil.

Paradoxalmente, a taxa de TxRDV no país tem diminuído, tendência agravada pela pandemia da COVID-19^6^. Embora indivíduos até o quarto grau de parentesco ou cônjuges possam doar sem restrições, doadores com vínculos mais distantes ou não aparentados necessitam de autorização judicial^11^. Contudo, essas restrições legais não afetam o sucesso do transplante, já que estudos demonstram resultados semelhantes entre doadores aparentados e não aparentados^9,12^.

Uma das principais barreiras ao TxRDV é a incompatibilidade imunológica, seja por incompatibilidade ABO (ABOi) ou prova cruzada positiva (PC+), que desqualificam cerca de 30%–50% dos potenciais doadores^7,13^. A DRP foi concebida para superar essas barreiras. Proposta inicialmente por Rapaport em 1986, a DRP permite que pares de doador e receptor incompatíveis troquem seus doadores, possibilitando transplantes que, de outra forma, seriam inviáveis^14^. Desde então, o método se expandiu mundialmente, com programas estabelecidos na Europa, Estados Unidos, Canadá, Austrália e outras regiões^15,16,17^.

Na América Latina, poucos países — incluindo o Brasil — relataram casos isolados de DRP, sendo o México aparentemente o único com um programa ativo^18,19,20^. Ao permitir cadeias mais longas e trocas complexas, a DRP pode aumentar significativamente as taxas de transplante, beneficiando pacientes sensibilizados que dificilmente encontrariam um doador compatível. Devido às características dos receptores incluídos nos programas de DRP, muitas vezes trocas envolvendo apenas dois pares não são suficientes para viabilizar os transplantes, sendo necessárias cadeias mais longas para atender pacientes altamente sensibilizados. Em 2006, um estudo evidenciou que um programa de DRP aumentou sua capacidade de transplante de 17,7% para 24,4% ao permitir trocas entre três pares, em vez de restringir-se a apenas dois^21^. Em 2010, outro estudo demonstrou que programas que aceitam trocas com N pares podem aumentar a capacidade de transplante em 18%, elevando também o Painel de Reatividade a Antígenos (PRA) médio dos receptores transplantados em 9%, em comparação com programas limitados a duplas^22^.

Essa solução inovadora tem grande potencial para o cenário de transplantes no Brasil^23^. Entretanto, a implementação de um programa nacional de DRP exigirá esforços logísticos e regulatórios significativos. O Brasil já vivenciou casos isolados de DRP, mas ainda carece de um programa nacional estruturado. Este relato descreve a primeira doação renal pareada envolvendo três duplas realizada no país, na Santa Casa de Misericórdia de Juiz de Fora (SCMJF), representando a experiência clínica inicial com essa modalidade no sistema brasileiro de transplantes.

O caso demonstra o potencial da DRP para aumentar as taxas de transplante e reduzir custos assistenciais, ao mesmo tempo em que destaca os desafios logísticos, regulatórios e éticos que precisam ser superados para uma implementação mais ampla. O sucesso dessa troca entre três duplas reforça a viabilidade da DRP como solução sustentável para a escassez de doadores renais no Brasil, pavimentando o caminho para o desenvolvimento futuro de um programa nacional de DRP.

## Ética

O projeto de pesquisa foi inicialmente aprovado pelo Comitê de Ética em Pesquisa do Hospital das Clínicas da Universidade de São Paulo (HCFMUSP) em 2018, como um estudo de centro único (CAAE: 83469518.4.0000.0068). Em 2022, uma emenda foi aprovada pelo mesmo comitê, ampliando-o para um estudo multicêntrico (CAAE: 83469518.4.0000.0068), e também pelo Comitê de Ética da Santa Casa de Misericórdia de Juiz de Fora (SCMJF) (CAAE: 83469518.4.2001.5139). Este relato de caso foi igualmente aprovado pelo Comitê de Ética em Pesquisa da SCMJF, em conformidade com as diretrizes éticas nacionais e internacionais para pesquisa envolvendo seres humanos (CAAE: 93197825.0.0000.5139).

Desde setembro de 2022, em uma parceria sem fins lucrativos com a *Alliance for Paired Kidney Donation*, passou a ser utilizada a ferramenta *KidneyMatchGrid* para inclusão de pares receptor-doador e realização de rodadas de compatibilidade (nas quais o sistema busca possíveis combinações entre os pares cadastrados). Em dezembro de 2023, foi identificada a possibilidade de uma troca envolvendo três duplas da mesma instituição (SCMJF).

O projeto cumpriu todos os requisitos legais brasileiros, seguindo um processo sequencial de aprovações, que incluiu: autorização do comitê de ética em pesquisa, aprovação judicial e subsequente autorização pelos Sistemas Estadual e Nacional de Transplantes. O processo judicial foi conduzido por um advogado vinculado ao projeto, financiado pela própria iniciativa, que gerenciou todos os trâmites legais de forma a evitar qualquer contato entre as duplas participantes, garantindo, assim, a preservação do anonimato e o cumprimento das exigências éticas.

### Pares Originais (Tabela 1)

**Tabela 1:** Dados Demográficos

Doadores			
	D1	D2	D3
Sexo	Feminino	Masculino	Feminino
Etnia	Branca	Branca	Parda
Idade	48	47	57
IMC	28,5	27,9	25,0
Tipo sanguíneo	A	A	O
TFGe	92	141	119
Relação	Filha	Marido	Irmã
Receptores			
	R1	R2	R3
Sexo	Masculino	Feminino	Feminino
Etnia	Parda	Parda	Parda
Idade	69	44	53
IMC	18,4	21,9	17,2
Tipo sanguíneo	O	A	A
cPRA	00%	69,5%	93,8%
Primeiro Tx	Sim	Sim	Não
Causa DRC	Hipertensão	Indeterminada	Indeterminada
Tempo em diálise (m)	10	18	12

Receptor 1 (R1), sexo masculino, 69 anos, em hemodiálise (HD) desde janeiro de 2023 devido a nefropatia hipertensiva, grupo sanguíneo O. Encontrava-se inscrito para transplante renal com doador falecido (TxDF) desde outubro de 2023, apresentando PRA de 0% para as classes I e II. Sua filha, doadora 1 (D1), 48 anos, grupo sanguíneo A, era candidata à doação, mas apresentava ABOi. A dupla R1–D1 foi incluída no programa de DRP em novembro de 2023.

Receptora 2 (R2), sexo feminino, 44 anos, em HD desde agosto de 2022 devido à DRC de etiologia indeterminada, grupo sanguíneo A. Inscrita para TxDF desde janeiro de 2023, apresentava Painel de Reatividade a Antígenos Calculado (cPRA) de 69,5%. Seu marido, doador 2 (D2), 47 anos, grupo sanguíneo A, era candidato à doação, porém foi contraindicado por PC+. A dupla R2–D2 foi incluída no programa de DRP em janeiro de 2023.

Receptora 3 (R3), sexo feminino, 53 anos, em HD desde agosto de 2022 por DRC de etiologia indeterminada, grupo sanguíneo A. Já havia sido submetida a um TxRDV entre 2004 e 2022. Foi inscrita novamente para transplante em junho de 2023, apresentando cPRA de 93,7%. Sua irmã, doadora 3 (D3), 57 anos, grupo sanguíneo O, foi contraindicada por PC+. A dupla R3–D3 foi incluída no programa de DRP em setembro de 2023.

### Compatibilização

A ferramenta *KidneyMatchGrid* identificou compatibilidade entre D1 e R2, D2 e R3 e D3 e R1 (Figura 1). O número de *mismatches* HLA entre as duplas pareadas variou de três a cinco *loci* (média de quatro), representando compatibilidade aceitável para o prosseguimento ao transplante. A compatibilidade foi posteriormente confirmada por citometria de fluxo negativa. Apesar do perfil sensibilizado dos receptores envolvidos, apenas R3 apresentou anticorpo específico contra o doador (DSA), identificado como Cw16 (MFI 1.584) no soro atual e DQ8 (MFI 1.628) no soro histórico. Nenhuma das duplas apresentou incompatibilidade relacionada ao risco infeccioso.

**Figura 1:** Doação pareada envolvendo 3 duplas.

Conforme descrito, o projeto foi aprovado pelos comitês de ética de ambos os hospitais participantes. Quando um potencial pareamento era identificado, a autorização era solicitada inicialmente ao Comitê de Ética do hospital, seguida de aprovação judicial — exigida pela legislação brasileira para doações entre não aparentados — e, por fim, autorização dos Sistemas Estadual e Nacional de Transplantes. Esse processo burocrático é demorado, evidenciando uma das principais dificuldades para a implementação prática de programas de DRP. Alterações legais — como o projeto de lei atualmente em tramitação na Câmara dos Deputados^24^ — parecem essenciais para viabilizar a criação de um programa nacional efetivo de DRP.

### Cirurgia

As seis cirurgias foram realizadas em 10 de agosto de 2024, na Santa Casa de Misericórdia de Juiz de Fora (SCMJF). Foram utilizadas seis salas cirúrgicas simultaneamente, iniciando-se pelos procedimentos dos doadores, seguidos imediatamente pelas cirurgias dos receptores correspondentes. Cada equipe era composta por quatro cirurgiões, anestesiologistas e residentes de cirurgia. Esse tipo de operação simultânea demanda amplo contingente de profissionais, coordenação precisa e infraestrutura hospitalar robusta. A discussão sobre a dessincronização das cirurgias, aplicável a cadeias maiores, está fora do escopo deste estudo.

O anonimato foi mantido antes e durante todas as etapas do processo de DRP. As avaliações pré-transplante e os exames laboratoriais foram agendados separadamente para evitar contato entre os participantes. No dia da cirurgia, doadores e receptores foram internados em andares distintos e levados ao centro cirúrgico em momentos diferentes. Os participantes foram informados de que eventuais encontros pós-transplante ocorreriam somente mediante consentimento mútuo, conforme especificado no Termo de Consentimento Livre e Esclarecido. Com a autorização de todas as partes, as apresentações foram feitas após os procedimentos cirúrgicos.

As cirurgias seguiram os protocolos institucionais de rotina e transcorreram sem complicações. Todos os receptores receberam terapia de indução com timoglobulina (2,25 mg/kg), com exceção da receptora com DSA detectado, que recebeu 6 mg/kg. O esquema de imunossupressão de manutenção consistiu em tacrolimo, sirolimo e prednisona para todos os pacientes. De acordo com os critérios institucionais, a elegibilidade dos doadores exigia depuração de creatinina > 80 mL/min, exame de urina normal e ausência de comorbidades ou contraindicações anatômicas. Todos os doadores tiveram alta no 2º dia pós-operatório (DPO 2), e todos os receptores, no DPO 4. Todos os receptores apresentaram função imediata do enxerto, com creatinina sérica entre 0,8 e 1,0 mg/dL na alta hospitalar.

Após um ano de acompanhamento, todos os receptores mantinham excelente função do enxerto, conforme ilustrado na Figura 2. O R1 apresentou queda transitória da função renal por problema prostático, sem relação com o enxerto, e evoluiu bem após a resolução, beneficiando-se da alta qualidade do rim doado por doador vivo. O mesmo ainda desenvolveu diabetes mellitus pós-transplante, mas, fora isso, não houve outras intercorrências ou reinternações. Nenhum dos pacientes apresentou suspeita de rejeição ou necessidade de biópsia por qualquer motivo. A R3 manteve o monitoramento do DSA, que permaneceu com MFI < 1000 durante todo o seguimento.

**Figura 2:** Creatinina dos receptores (mg/dL).

Todos os doadores também apresentaram recuperação pós-operatória adequada, sem complicações no primeiro ano após a doação, e mantiveram função renal satisfatória, com TFGe entre 58 e 83 mL/min.

## Discussão

A primeira experiência com Doação Renal Pareada (DRP) no Brasil ocorreu em março de 2020, no Hospital das Clínicas da Faculdade de Medicina da Universidade de São Paulo (HCFMUSP), envolvendo uma troca entre dois pares com ABOi, realizada com sucesso^19^. Em 2022, publicamos um artigo discutindo os potenciais benefícios da DRP e propusemos que o tema fosse trazido para uma discussão mais ampla na sociedade^25^. O editorial que acompanhou essa publicação reforçou as vantagens da DRP, respaldadas pela literatura existente, enfatizando que pouco ou nenhum impacto social ou político negativo foi observado em decorrência da implementação de programas de DRP em outros países. Considerando que o transplante renal é a melhor terapia para o tratamento da DRC, a doação pareada deve ser considerada para todos os pacientes que possuam doadores vivos potenciais, mas incompatíveis^26^. No Congresso Americano de Transplantes (ATC) em 2024, apresentamos um levantamento com 89 candidatos à doação renal em vida na SCMJF, mostrando que 70% desconheciam a DRP, mas 80% expressaram disposição em participar do programa^27^.

O custo da diálise no Brasil é extremamente alto^28^, sendo o transplante renal o tratamento mais custo-efetivo, além de seus demais benefícios amplamente reconhecidos. O TxRDV oferece melhores taxas de sobrevida em comparação ao transplante renal com doador falecido (TxDF), tornando-se também mais vantajoso economicamente^16,29^. Um estudo nacional publicado em 2016 estimou que o TxDF gera uma economia por paciente de R$ 37.000 a R$ 74.000 em comparação à hemodiálise (HD) e à diálise peritoneal (DP), respectivamente, enquanto o TxRDV proporciona economias ainda mais expressivas, de R$ 46.000 e R$ 82.000^30^.

Para o êxito da implementação nacional de um programa de DRP, o Brasil, assim como outros países em desenvolvimento, deve enfrentar diversos desafios estruturais e regulatórios^31^. Entre esses desafios estão o desenvolvimento de uma infraestrutura logística sólida, a simplificação de processos burocráticos complexos e a destinação de recursos financeiros sustentáveis em um país de dimensões continentais. O processo de aprovação em múltiplas instâncias, exigido para cada caso de DRP, ainda é demorado e representa um dos principais entraves operacionais à expansão da iniciativa. Assim, atualizações legais e regulatórias — como o projeto de lei atualmente em discussão na Câmara dos Deputados^24^ — serão provavelmente essenciais para permitir o estabelecimento de um programa nacional permanente e eficiente. Considerando que o sistema de transplantes brasileiro é público e centralizado, a criação de um programa de DRP só fará sentido se for organizada como uma iniciativa única, coordenada pelo Ministério da Saúde por meio do Sistema Nacional de Transplantes, garantindo equidade de acesso e supervisão ética unificada.

Para um programa multicêntrico eficaz, a experiência internacional demonstra que o transporte de órgãos é a abordagem mais eficiente^32,33^, por permitir que doadores e receptores permaneçam em seus centros de origem, sob os cuidados de suas equipes de confiança. No Brasil, a aviação comercial é rotineiramente utilizada para o transporte de órgãos provenientes de doadores falecidos, e acreditamos que, com a devida autorização, essa estratégia também seria ideal para programas de DRP. No entanto, uma discussão detalhada dessa logística extrapola o escopo do presente estudo. Além disso, é necessário investir no desenvolvimento tecnológico e na capacitação de recursos humanos dos laboratórios de imunologia. Igualmente importante é a disseminação de informações precisas, tanto na comunidade médica quanto na sociedade em geral, a fim de desmistificar preconceitos, especialmente em relação à doação renal entre não aparentados e à doação renal não direcionada.

Embora apenas uma das três receptoras deste caso fosse altamente sensibilizada, a inclusão de um conjunto mais amplo de duplas receptor–doador está em consonância com as práticas internacionais da DRP, já que a diversidade dentro do grupo é essencial para alcançar compatibilizações viáveis e expandir o acesso aos pacientes com maiores desafios imunológicos. Com base em um modelo preditivo apresentado no Congresso Mundial de Transplantes de 2025^34^, a probabilidade estimada de transplante em um ano para esses pacientes, caso dependessem apenas da lista de TxDF, seria de 40,6% para R1, 35,1% para R2 e apenas 9,7% para R3. O mesmo modelo estimou tempos médios de espera de 17 meses para R1, 21 meses para R2 e ausência de previsão de transplante em até 120 meses para R3. Por meio do programa de DRP, esses pacientes não apenas alcançaram os desfechos superiores associados ao TxRDV, mas também o fizeram em um intervalo de tempo significativamente menor. Para R3, em particular, a participação no programa de DRP provavelmente representou sua única oportunidade realista de retransplante.

No Brasil, pacientes com doadores vivos saudáveis e dispostos, mas incompatíveis imunologicamente, permanecem na mesma condição daqueles sem qualquer doador, enfrentando possibilidades limitadas de transplante. Essa situação afeta não apenas os receptores, que permanecem em diálise com baixa expectativa de transplante, mas também os doadores, privados da oportunidade de concretizar seu desejo de doar e ajudar seus familiares. A DRP otimiza o uso de um recurso escasso e valioso — o doador vivo saudável e motivado — e, ao fazê-lo, tem o potencial de aumentar o número total de transplantes, melhorar a sobrevida de enxertos e receptores e prevenir o tráfico de órgãos, uma prática ilegal e amplamente condenada pela sociedade brasileira e pela comunidade transplantadora^35,36^.

As principais barreiras para a implementação de um programa nacional de DRP no Brasil incluem a insegurança jurídica, a logística complexa e a falta de integração institucional entre os centros transplantadores. A implementação ampla exigirá reforma legislativa, coordenação nacional pelo Ministério da Saúde e investimento contínuo em capacitação e infraestrutura para garantir equidade e transparência.

## Agradecimentos

Gostaríamos de expressar nossa gratidão aos professores Michael Rees e Alvin Roth pelo desenvolvimento e pela disponibilização gratuita da plataforma *KidneyMatchGrid*. Seu apoio técnico e científico inestimável foi fundamental para a implementação bem-sucedida do programa de doação renal pareada no Brasil e para a realização dos transplantes relatados neste estudo.

## Data Availability

Todo o conjunto de dados que dá suporte aos resultados deste estudo está disponível mediante solicitação ao autor correspondente Juliana Bastos. O conjunto de dados não está publicamente disponível por conter dados clínicos sensíveis extraídos de prontuários médicos e dados parciais de um projeto de pesquisa de doutorado ainda em andamento, mas pode ser disponibilizado mediante solicitação justificada ao autor correspondente, respeitando as normas éticas e legais vigentes.
